# Cork oak and climate change: Disentangling drought effects on cork chemical composition

**DOI:** 10.1038/s41598-020-64650-9

**Published:** 2020-05-08

**Authors:** Carla Leite, Vanda Oliveira, Isabel Miranda, Helena Pereira

**Affiliations:** Centro de Estudos Florestais, Instituto Superior de Agronomia, Universidade de Lisboa, Tapada da Ajuda, 1349-017 Lisboa, Portugal

**Keywords:** Drought, Abiotic, Plant sciences, Plant stress responses

## Abstract

Climate change induces in the Mediterranean region more frequent and extreme events, namely, heat waves and droughts, disturbing forest species and affecting their productivity and product quality. The cork oak (*Quercus suber*) is present along the western Mediterranean basin and its outer bark (cork) is sustainably collected and used for several products, mainly wine bottle stoppers. Since most cork properties arise from its chemical composition, this research studies the effect of drought on cork chemical composition (suberin, lignin, polysaccharides and extractives) and on polysaccharide and suberin monomeric composition. Three sets of cork samples, from the same site, were examined: in one set the cork grew without drought; in another two drought events occurred during cork growth and in the third one drought event happened. The results show that, in general, drought does not affect the proportion of the main components of cork, the monomers of suberin or of polysaccharides, with few exceptions e.g. drought increased ethanol extractives and xylose in polysaccharides and decreased arabinose in polysaccharides. The variability associated to the tree is much more relevant than the effect of drought conditions and affects all the parameters analyzed. Therefore, our research suggests that the tree genetic information, or its expression, plays a much more important role on the chemical composition of cork than the drought conditions occurring during cork growth. In practical terms, the potential increased occurrence of droughts arising from climatic changes will not compromise the performance of cork as a sealant for wine bottles.

## Introduction

Climate change is happening and several authors agree that forest species are already being affected (e.g^1–3^.) and will keep on being in the future^4–8^. The intensity and the way species are affected depends on the dimension of their present and future distribution area, their environmental tolerance, and capacity to disperse^9^ while the scientific community and forest managers may have an active role in adapting and mitigating climate changes effects.

The Mediterranean region is considered to be a hotspot for climate change^10^ for which the models predict an increase in the temperature and a pronounced decrease in the precipitation^10–13^, corresponding to an intensification in frequency, intensity and duration of drought, mainly during the warm season^7,11,14,15^. Consequently, this region will experience high water stress conditions and reduced vegetation production.

The cork oak (*Quercus suber*) is an evergreen oak species distributed along the western Mediterranean basin, covering an area of approximately 2.1 million ha^16^. Its outer bark (cork) is periodically (usually every 9 years) removed on a sustainable procedure, corresponding to an annual production of up to 200 thousand tons^16^. This forest product is the second most important non wood forest product commercially exploited^11^ and it is the raw material of an important industry with diversified products and applications, from sealants to agglomerates and composites used as insulation materials, surfacing panels for construction and aeronautics, pollutants absorbers, clothing and decorative articles, and the one with the most added value – cork stoppers for the wine industry, corresponding to 70% of the generated income of the cork industry^17–20^.

Most of the characteristics of cork, namely high compressibility, flexibility under compression, low permeability and chemical and biological inertness come from its chemical composition^18,21^. Actually, many of the cork properties arise from the presence in cork cell walls of its main component – suberin^18,21^. Besides suberin (that represents on average 43%), cork is also constituted by lignin (22%), polysaccharides (19%) and extractives (16%)^19^. The monomeric composition of suberin was object of several analysis with different analytical methods (see^18^ for a review on this subject). ^22^ identified ω-hydroxyacids (31% of the total monomers), α,ω-diacids (53%), *n-*alkanols (<1%) and ferulic acid (<1%) (values calculated by^23^). The holocellulose of cork is mainly composed of glucose (50% of total monosaccharides) and xylose (35%), with smaller amounts of arabinose, mannose, galactose and rhamnose^21^. Nevertheless, there is a substantial variability in the chemical composition of *Q. suber* cork regarding between-tree and between site differences that has been evaluated in several research works^19,24–28^.

The impact of drought on cork growth was addressed in numerous studies (e.g^29–34^.) concluding that drought severely reduces cork growth but cork oak is very resilient and cork growth rapidly recovers when drought conditions end. To date and to our knowledge, there are no studies on the effect of drought on cork chemical composition. The objective of this study is to analyze the effect of drought on cork chemical composition. Our specific goals are to examine if drought induces any changes in the proportion of the main chemical constituents of cork and in the suberin and polysaccharide monomeric composition that could compromise the utilization of cork for the production of wine stoppers.

## Material and Methods

### Material

The cork samples used in this research were collected in a cork oak stand (*montado*) located in central west Portugal, in the Coruche municipality, inside the region that is considered to be one of the best production regions for the cork oak. The site has around 190 ha with 67 trees/ha and is located in the Tagus river basin where Mediterranean climate is influenced by the Atlantic Ocean.

The samples were randomly collected at breast height (1.3 m above ground), during the cork stripping season, in mature trees under exploitation. The analyzed samples were collected with different drought conditions in their timespans of the cork production cycle: 10 samples were collected in a timespan without any drought, 10 samples in a period when one drought occurred and 10 samples with two droughts.

Table 1 shows a brief description of the climatic framework for the samples - the years of cork striping (harvesting), the years of the occurrence of the droughts, the respective annual Standard Precipitation Evapotranspiration Index (SPEI) and the drought classification according to^35^. The Standard Precipitation Evapotranspiration Index (SPEI), developed by^36^, is one of the most used indicators for drought analysis and droughts in the Iberian Peninsula are better detected with SPEI than with other indices like the Standard Precipitation Index (SPI)^35^. Furthermore, the classification of droughts proposed by^35^ and followed by^37^ categorizes drought indices according to four classes: no drought if SPEI > −0.84; moderate if −0.84 > SPEI > −1.28; severe, if −1.28>SPEI > −1.65 and extreme, if −1.65 > SPEI.

**Table 1:** Cork stripping years, years with drought, annual SPEI and drought classification of drought years according to^35^.

Stripping year	Years with drought	Annual SPEI of the drought year	Drought classification
1994	—	—	—
2003	1995	−1.22	Moderate
	1999	−1.54	Severe
2012	2005	−2.22	Extreme

The 30 samples (with approximately 15 × 15 cm^2^) were boiled in water for one hour at atmospheric pressure and left to air-dry until equilibrium, in a procedure similar to the one usually performed by the cork industry for the production of cork stoppers. Each sample was cut in small pieces with a chisel and the back (outermost bark layer of phloemic tissues) and the belly (innermost cork layer) removed to avoid contamination with other materials. The small pieces of cork were first milled with a knife mill (Retsch SM 2000) passing through a 2 × 2 mm^2^ sieve and, afterwards, with an ultra-centrifugal mill (Retsch ZM 200). The material was granulometric separated with a vibratory sieve (Retsch AS 200basic) for 10 minutes. The fractions that passed the 60 mesh screen (0.250 mm) were discarded to avoid contamination with lenticular material or woody inclusions that are chemically different from cork and the particles between 40 (0.425 mm) and 60 mesh were used, as usually performed for cork chemical analysis^21^.

### Chemical analysis

Summative chemical composition comprised the determination of extractives, suberin, Klason and acid-soluble lignin and the monomeric composition of polysaccharides. The analytical procedures were previously described by^38^ and are here only briefly detailed. Extractives content was determined by successive Soxhlet extractions of cork samples with dichloromethane (6 h), ethanol (16 h) and water (16 h). The suberin content was determined in the extractive-free cork using methanolysis for depolymerization^38^. The suberin content (that corresponds to the fatty acids and alcohols derivatives resulting from suberin depolymerization) was quantified as percent of dry cork mass.

Klason and acid-soluble lignin were determined on the pre-extracted and desuberinized material using total hydrolysis with sulphuric acid.

The polysaccharides content was determined by quantification of the monosaccharide monomers released by the total acid hydrolysis used for lignin determination^38^. The sugar monomers were determined using a high-performance anion exchange chromatography (HPAEC) using Aminotrap plus CarboPac SA10 anion exchange columns. The carbohydrate composition was expressed in percent of total monosaccharides.

For each tree, extractives analysis was performed in 3 independent (sub)samples collected in the fraction of particles between 40 (0.425 mm) and 60 mesh (triplicate aliquots), determination of suberin and lignin were done in duplicate aliquots, and monomeric composition of polysaccharides was determined in one sample/tree.

The monomeric composition of suberin was determined in aliquots from the methanolic extracts obtained after the depolymerization of suberin^38^. The samples were evaporated, derivatized by trimethysilylation and immediately analyzed by GC-MS, with the following Zebron conditions: Zebron 7HGG015-02 column (Phenomenex, Torrance, CA, USA) (30 m, 0.25 mm; ID, 0.1 µm film thickness), injector 400 °C, oven temperature program: 50 °C (held 1 min), 10 °C min^-1^ to 150 °C, 5 °C min^-1^ to 200 °C, 4 °C min^−1^ to 300 °C, 10 °C min^-1^ to 380 °C (held 5 min). The MS source was kept at 220 °C and the electron impact mass spectra (EIMS) taken at 70 eV of energy. The experimental procedure used for the suberin compositional determination does not allow the quantification of glycerol but only of the long-chain fatty components.

### Statistical analysis

All results were expressed as mean and standard deviation.

To evaluate the effect of the occurrence of drought(s) on the chemical composition of cork, a mixed model approach was used^39^, considering the stripping year (that is directly connected to the number of droughts that occurred in the growing period of the cork - Table 1) as a factor of fixed effect and the tree, nested on the stripping year, as a factor with random effect. With this approach the variability associated to the tree is accounted for and, therefore, we can: (i) better estimate the (fixed) effect of the drought on the chemical composition and (ii) evaluate if there is variability associated to the tree. For this analyses the R package *nlme* was used^40,41^ specifying the maximum likelihood as the fitting method, as it is the only method that allows the estimation of the fixed effects estimators. To validate the underlying distributional model assumptions^39^, namely normality of the residuals and of the predictors of random effects, independence and homogeneity of variances, we used the traditional plots (trough the commands *plot* and *qqnorm)*, because they are considered to be the most useful methods for assessing the validity of the abovementioned assumptions^39^.

For the monomeric analysis of the polysaccharides and of the suberin, an analysis of variance was performed. In this analysis the variability associated to the tree could not be accounted. The normality assumption for all the variables was confirmed with the Shapiro-Wilk test and the equality of variances validated with a F test. Whenever these tests failed, a non-parametric approach was used with the Kruskal-Wallis rank sum test and if differences occurred the Wilcoxon test was also applied. This statistical analysis was performed using the R programming language^41^. In all the statistic procedures the effects were considered as statistically significant when the *p*-value was less than or equal to 0.05.

## Results

The summative chemical composition of the cork samples produced without drought and with one and two drought events during the cork growth period is shown in Table 2. The mean content of total extractives is 12.1% (no drought), 12.5% (one drought) and 12.1% (two droughts). The ethanol and water-soluble compounds accounted for about 60% of the total extractives and non-polar compounds soluble in dichloromethane for about 40%. Suberin content ranged between 36.0% (one drought) and 38.2% (two droughts), and total lignin between 27.9% (no drought) and 26.0% (two droughts).

**Table 2:** Chemical composition (mean and standard deviation) of the cork samples according to the year of debarking (and number of droughts), in % of total dry mass.

Chemical parameter	1994 (no drought)	2003 (two droughts)	2012 (one drought)
**Extractives total**	12.05 ± 1.79	12.09 ± 1.22	12.52 ± 1.70
Dichloromethane	5.18 ± 1.04	5.33 ± 0.66	4.93 ± 0.82
Ethanol	3.00 ± 1.06	3.03 ± 0.99	4.12 ± 1.23
Water	3.87 ± 0.67	3.73 ± 0.69	3.47 ± 0.72
**Suberin**	36.56 ± 3.32	38.24 ± 3.67	35.97 ± 4.50
**Lignin, total**	27.93 ± 2.38	26.04 ± 2.71	27.74 ± 3.30
Klason	26.63 ± 2.36	24.72 ± 2.75	26.54 ± 3.18
Acid soluble	1.30 ± 0.26	1.32 ± 0.19	1.19 ± 0.24
**Ratio suberin/total lignin**	1.33 ± 0.22	1.49 ± 0.27	1.33 ± 0.33
**Polysaccharide composition (% of total monosaccharides)**			
Rhamnose	1.48 ± 0.50	0.82 ± 0.19	1.35 ± 0.47
Arabinose	19.22 ± 4.25	14.19 ± 2.41	17.82 ± 2.57
Galactose	8.92 ± 2.03	6.31 ± 0.79	8.04 ± 0.77
Glucose	40.73 ± 2.81	40.22 ± 0.96	39.24 ± 1.57
Xylose	20.11 ± 6.16	29.64 ± 3.79	23.78 ± 1.91
Mannose	1.95 ± 3.11	1.12 ± 1.20	1.59 ± 1.97
Galacturonic acid	5.73 ± 1.51	4.45 ± 0.36	5.89 ± 0.93
Glucuronic acid	1.18 ± 1.60	2.47 ± 0.16	1.11 ± 1.44
Acetyl	0.68 ± 0.22	0.78 ± 0.38	1.19 ± 0.76

Monosaccharide composition is expressed in % of total monosaccharides.

The mixed model analysis, performed with maximum likelihood, revealed that the year of debarking i.e. the number of drought events during the cork growth period did not have a significant effect on the chemical composition of cork, except in the content of extractives soluble in ethanol (p-value = 0.03), that was significantly higher in 2012 (see Table 2 and supplementary material).

Concerning the variability associated to the tree, all the chemical parameters (extractives, suberin and lignin) showed a significant variability (p-values between 1.4 × 10^−3^ and <2.0 × 10^−16^), meaning that the tree has a much more significant effect on the proportion of the chemical parameters than the drought conditions during the cork growth.

The mixed model assumptions were all confirmed as the performed graphics showed the “confirming” shapes and no outliers of random effects were seen. The random effects and residual variances are presented as supplementary material (Table S2). Their estimate was not our primary interest – we actually wanted to evaluate if the effect of the fixed factor (drought) was significant on the cork chemical composition and if there was variability associated to the tree. Therefore, the mixed model approach was used mainly to account for the variability associated to the random factor (tree) in the statistical analysis (which cannot be done with other linear models like the ANOVA as this variability is included in the error). Regarding the carbohydrate composition in proportion of the total monomers, glucose ranged between 39.2% (one drought) and 40.7% (no droughts), xylose between 20.1% (no drought) and 29.6% (two droughts), and arabinose between 14.2% (two droughts) and 19.2% (no drought). The cork polysaccharides also contained smaller amounts of other monomers: on average 1.2% rhamnose, 7.7% galactose, 1.6% mannose, 5.4% galacturonic acid, 1.6% glucuronic acid and 0.9% acetyl groups.

The analysis of variance showed that there is a significant effect of the drought conditions on the xylose and arabinose contents (p-value=1 × 10^−5^ and 5 × 10^−3^ respectively). Cork produced under two drought events had higher amounts of xylose and lower levels of arabinose than the cork produced without or with one drought event (p-values = 1.0 × 10^−3^/1.0 × 10^-4^ and 4.3 × 10^−3^/4.0 × 10^−3^ respectively) that are not different from each other. The glucose amounts didn’t follow a normal distribution (p value= 0.04) neither the ratios between glucose and xylose and between glucose and the sum of xylose and arabinose (p-values of 8.0 × 10^−4^ and 5.8 × 10^−5^, respectively). The non-parametric test revealed that glucose and the ratio between glucose and the sum of xylose and arabinose were not affected by drought but the ratio glucose/xylose showed an unclear pattern since cork debarked in 2012 had the highest ratio and cork grown under two drought events the lowest ratio (p-values between 0.02 and 7.5 × 10^−4^). The amount of the other sugars did not seem to be affected by drought.

A graphical representation of most of the chemical parameters summarized in Table 2 can be found as supplementary material.

### Suberin composition

The monomeric composition of the suberin of the samples produced without drought (1994), with two drought events (2003) and one drought event (2012) is presented in Table 3, in percent of the peak area in relation to the total peak chromatogram area, grouped by chemical families. The detailed composition by monomer is shown as supplementary data. The monomers identified by GC-MS were the same in all the samples.

**Table 3:** Composition (by chemical family) of suberin from cork produced without any drought (1994), with two drought events (2003) and one drought event (2012), determined in the GC–MS chromatograms of the depolymerization extracts (percentual peak area and standard deviation).

**Identified families**	**1994** **(no drought)**	**2003** **(two droughts)**	**2012** **(one drought)**
Alkanoic acids saturated	6.50 ± 1.46	7.79 ± 1.31	7-90 ± 1.60
Alkanoic acids with mid-chain substitution	17.34 ± 2.12	18.04 ± 1.36	17.41 ± 1.92
**(Total alkanoic acids)**	**23.83** ± **1.54**	**25.83** ± **1.36**	**25.31** ± **0.64**
Alkanoic α,ω-diacids saturated	14.37 ± 1.35	12.18 ± 1.31	12.95 ± 1.52
Alkanoic α,ω-diacids with mid-chain substitution	4.59 ± 0.94	3.35 ± 0.61	5.10 ± 1.10
**(Total alkanoic α,ω-diacids)**	**18.95** ± **1.58**	**15.53** ± **1.81**	**18.06** ± **2.58**
ω-Hydroxyl alkanoic acids saturated	19.02 ± 1.28	16.37 ± 1.50	17.18 ± 2.51
ω-Hydroxyl alkanoic acids with mid-chain substitution	24.05 ± 1.46	21.37 ± 2.05	24.19 ± 1.48
**(Total** ω**-hydroxyl alkanoic acids)**	**43.07** ± **2.50**	**37.74** ± **1.92**	**41.37** ± **3.52**
Alkanols	1.99 ± 0.30	2.84 ± 0.60	2.44 ± 0.57
Aromatics	2.12 ± 0.29	2.43 ± 0.53	2.10 ± 0.74
Sterols	0.10 ± 0.02	0.11 ± 0.01	0.08 ± 0.06
Glycerol and glycerides	1.81 ± 1.38	3.15 ± 1.04	2.73 ± 0.86
Terpenoids	0.67 ± 0.11	0.90 ± 0.14	0.62 ± 0.29
**Identified**	**92.55**	**88.53**	**92.71**
**Unidentified**	**7.45**	**11.47**	**7.29**

The main monomers were the ω- hydroxyl alkanoic acids (representing between 37.7% and 43.1% of the total), namely the ω-hydroxyl alkanoic acids with mid-chain substitution, representing between 21.4% and 24.2% of the total monomers found in the suberin depolymerization products. Alkanoic acids represented between 23.8% and 25.8% of the total monomers and were mainly mid chain substituted. Alkanoic diacids ranged between 15.5% and 19.0% and showed a higher proportion of saturated acids. The other identified monomers presented much smaller values (e.g. 3.2% for glycerol and glycerides under two drought events).

The statistical analysis showed that there was no effect of drought on the proportion of the identified families of the suberin monomers.

## Discussion

This research focused on evaluating if drought events occurring during the cork production cycle (i.e. the years comprised between two cork strippings) affect the chemical composition of cork, namely, if they modify the relative proportion of the chemical constituents in a way that could compromise the utilization of cork for wine stoppers. In fact, cork chemical composition is directly associated to the material’s properties, namely to the permeation to gases and liquids and performance of cork stoppers in wine bottles^18^.The present study on the chemical composition variation of cork was designed to have representativeness of samples while the timespan allowed to use a temporal control^42^ and the use of a single site for the sampling reduced the effects of confounding variables rather than the drought. Also the sampling in all the cases at only one location within the tree (breast height) avoids any variability associated with a potential chemical variation along the cork oak stem^21^.

There are a few species in addition to the cork oak that produce considerable amounts of cork, as reviewed by^23^. Most of the scientific works about those species were performed recently and, as far as we know, none has addressed the effect of climate on their cork chemical composition. Also, the effect of drought on the chemical composition of wood has not gathered much attention among the scientific community. Nevertheless^43^, analyzed the effect of drought on the concentration of wood terpenoids in *Pinus sylvestris* and *Picea abies* seedlings, concluding that severe drought increased the concentration of several individual monoterpenes and resin acids (respectively 39 and 32% higher in Scots pine and 35 and 45% higher in Norway spruce). Therefore, drought may affect the chemical composition of secondary metabolites extractives, a study that was not made in present cork analysis.

Regarding the chemical composition of cork, it is known that there is a large natural variability^19^ that can, at least partially, contribute to understand the diversity in the behavior of cork products, particularly of cork stoppers^38^. Several studies addressed the chemical variation of cork^19,25–28,44^ but the emphasis of these studies has been on the analysis of the geographical variability rather than on the climate effect on the chemical composition. To our knowledge, this is the first research about the effect of drought on the chemical composition of cork.

Overall, the chemical composition of the cork samples that were analyzed (Table 2) are in the range of the results known for cork, as reported by several authors (as reviewed in^21^).

For instance, the average 12.2% extractives of which 40.0% are non-polar compounds soluble in dichloromethane (Table 2) are comprised in the range of values reported by e.g.^19,24,26,28^. The results show that drought does not seem to affect the total amount of extractives and that the variation that was found is more related with the genetic information of the tree.

This applies also to the non-polar extractives that are lipophilic compounds including long-chain fatty acids and alcohols, and triterpenes^19,21^ and are related to the permeability of cork with lower amounts inducing higher permeability. Our results suggest that drought does not affect this component and that it is the tree that accounts for the existing variation. This natural variability is well recognized and translates, for instance, in different oxygen transfer through cork stoppers into the wine bottles^45^.

Drought enhanced the amount of the polar extractives soluble in ethanol that may contribute to the organoleptic properties of the cork bottled wine. However, the tree was a much more significant factor for the variation of these extractives thereby overruling any drought induced changes in this wine characteristic.

The content of suberin is the most important chemical attribute of cork since it is its chemical fingerprint and directly related to most of its typical properties, namely those linked to the materials flexibility and hydrophobicity^18^. The mean values for suberin (36.0% to 38.2%, Table 2) are within the range of values reported by the existing studies (e.g^19,26,28^.).

Climate conditions, namely drought, do not seem to induce any changes in the proportion of suberin in cork; on the contrary, it is the tree genetic information that has a very strong impact on suberin content. In fact^46^, refer that suberin varies within the species according to its geographical location and the tree condition. Our samples were collected in the same site and the trees were all mature production trees in good phytosanitary conditions, therefore the high variability found in our results should come from the tree genetics.

Lignin is the second most abundant component of cork, giving mechanical support and rigidity to the cell walls^21^. Our samples contained mean values of total lignin between 26.0% and 27.9% (Table 2) which are in the range presented by^19^ and^26^. Drought had not a significant effect on Klason lignin, acid soluble lignin or total lignin contents, but the tree had a very strong influence on the relative amount of these compounds.

The proportion between suberin and lignin defines cork’s unique properties namely mechanical behavior, resilience and permeability^18^. Our samples showed a lower mean ratio suberin/lignin when compared to the mean results obtained by^19^ and^26^ but still within the range of their values. Drought did not influence the suberin-to-lignin ratio and again it is the tree genetics the important factor on the variation of this feature.

The monosaccharide composition of the cork samples (Table 2) is within the range of values reported for cork^18,28^. It should be highlighted that the ratio glucose/xylose was influenced by drought although without a clear pattern i.e. the cork produced under two droughts episodes (debarked in 2003) had significantly lower levels of glucose/xylose than the cork grown in the other two periods but the cork produced without drought had a lower ratio than the cork produced under one drought event. Arabinose content increased and xylose was negatively affected by drought but only if two droughts occur during the formation of the cork.

Regarding the monomeric composition of suberin obtained by GC-MS analysis (Table 3), our data shows that the main chemical families found were ω-hydroxyl alkanoic acids (37.7 to 43.1% of total monomers) especially with mid-chain substitution, representing between 24.1 and 24.2% of total monomers. Alkanoic α,ω-diacids (15.5 to 19.0%) and total alkanoic acids (23.8 to 25.8%) represented most of the remaining monomers. The proportion for the three main chemical families is somewhat different from that given by other authors, namely regarding the content in alkanoic acids^22^: reported 53.0% of α,ω-diacids, 30.6% of ω-hydroxyl alkanoic acids, and less than 2% of alkanoic acids, and^47^ referred 29.5% of α,ω-diacids, 52.9% of ω- hydroxyl alkanoic acids and less than 2% of alkanoic acids. This variation reinforces that there is a significant variability on the suberin monomeric composition, as already reported by^24^, possibly controlled by the genetic information of the tree.

In fact, our results suggest that the tree is more important in the chemical composition of cork than the wet conditions underlying the development of the cork. This conclusion is in line with reports that genetics must be a much more relevant factor in cork chemical composition and performance than other factors like geographical origin^19,21,25,26^. The chemical composition of cork produced under drought conditions is well within the variation range found by several authors for cork. Moreover, our results show that drought does not trigger the production of different compounds and has only a minor effect on the proportion of the chemical constituents of cork.

Therefore, the occurrence of drought events during the cork growth cycle does not seem to compromise the behavior of cork, namely when it is used as sealant material in wine bottles. However a word of caution must be given since cork properties arise not only from chemical composition but also from the cellular structure, namely cell dimensions^18,21^. With drought leading to thinner cork rings and less and smaller cells^33,34,48^, an analysis of the effect on cork cellular structure should be made in order to evaluate the full impact of drought on cork behavior. Nevertheless, the large variation found in cork ring width in commercial cork planks used for the production of wine stoppers^33,34^ allows to consider that this will not be a critical factor.

## Conclusions

It is well known that there is large natural variability on the chemical composition of cork but research has focused mostly on its geographic variability. This paper presents the first analysis on the climatic effects of drought on the chemical composition of cork, including the proportion and monomeric composition of the main chemical constituents and an experimental design allowing to discriminate the drought and the individual tree response.

The results show that drought has a negligible effect on the cork chemical constitution namely regarding its structural components proportion and composition, and specifically the suberin-to-lignin proportion and suberin composition that are the flagship characteristics of cork underlying its properties. The genetic package of the tree is the most important factor of chemical variation of cork that overrules any impact from drought conditions.

Therefore, the expected more frequent and severe forthcoming droughts in the Mediterranean region where the cork production areas are included will not compromise the cork properties related to its chemical composition, namely the performance of cork as a sealant for wine bottles.

## Supplementary information

Supplementary information.
