# Supplementary material for: Cork oak and climate change: Disentangling drought effects on cork chemical composition
Source: Sci Rep. 2020 May 8;10:7800. doi: 10.1038/s41598-020-64650-9 (PMC7210947; doi:10.1038/s41598-020-64650-9)
Supplement: Supplementary file 1 — Supplementary information. [file 41598_2020_64650_MOESM1_ESM.pdf]

Cork oak and climate change: Disentangling drought effects on cork chemical composition

Carla Leite, Vanda Oliveira, Isabel Miranda and Helena Pereira

Supplementary material

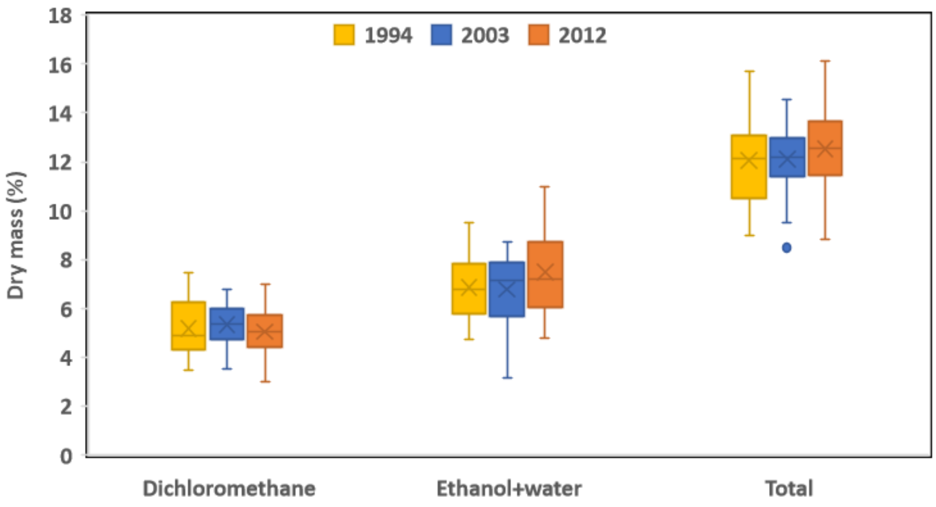

Figure S1 – Extractives content (in % of dry mass) of the samples grown without any drought (1994), with two drought events (2003) and one drought event (2012).

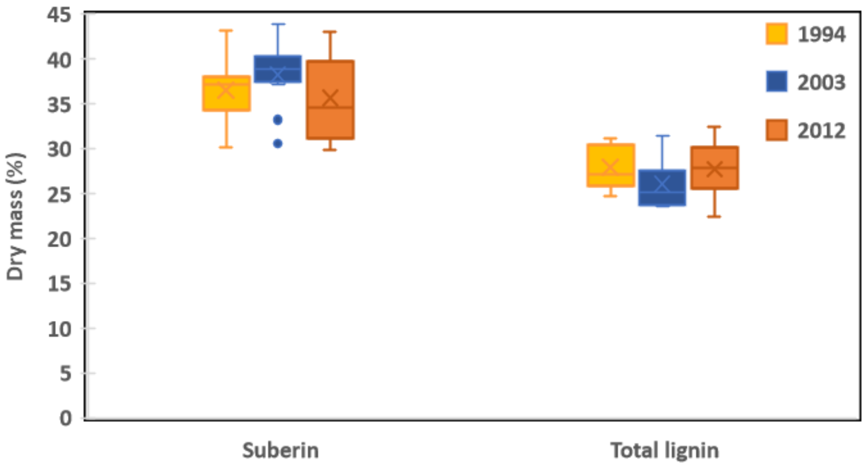

Figure S2 – Suberin and total lignin contents (in % of dry mass) of the samples grown without any drought (1994), with two drought events (2003) and one drought event (2012).

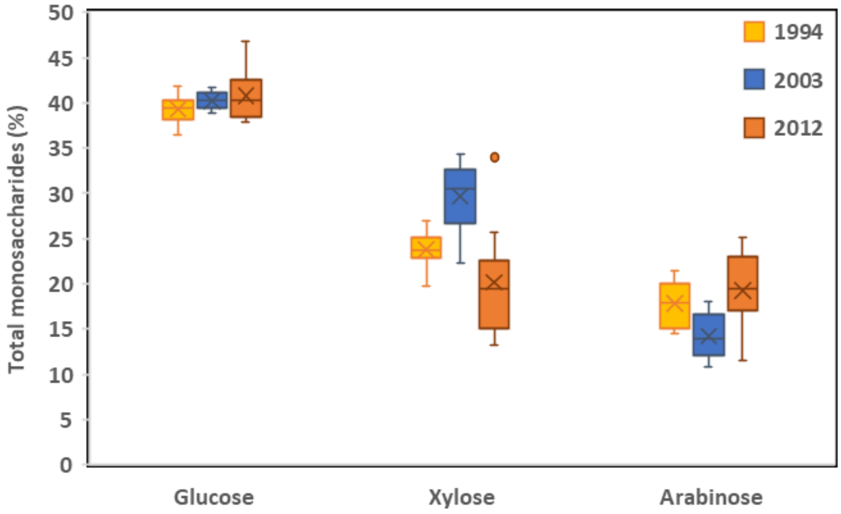

Figure S3 – Glucose, xylose and arabinose proportion (in % of total monosaccharides) of the samples grown without any drought (1994), with two drought events (2003) and one drought event (2012).

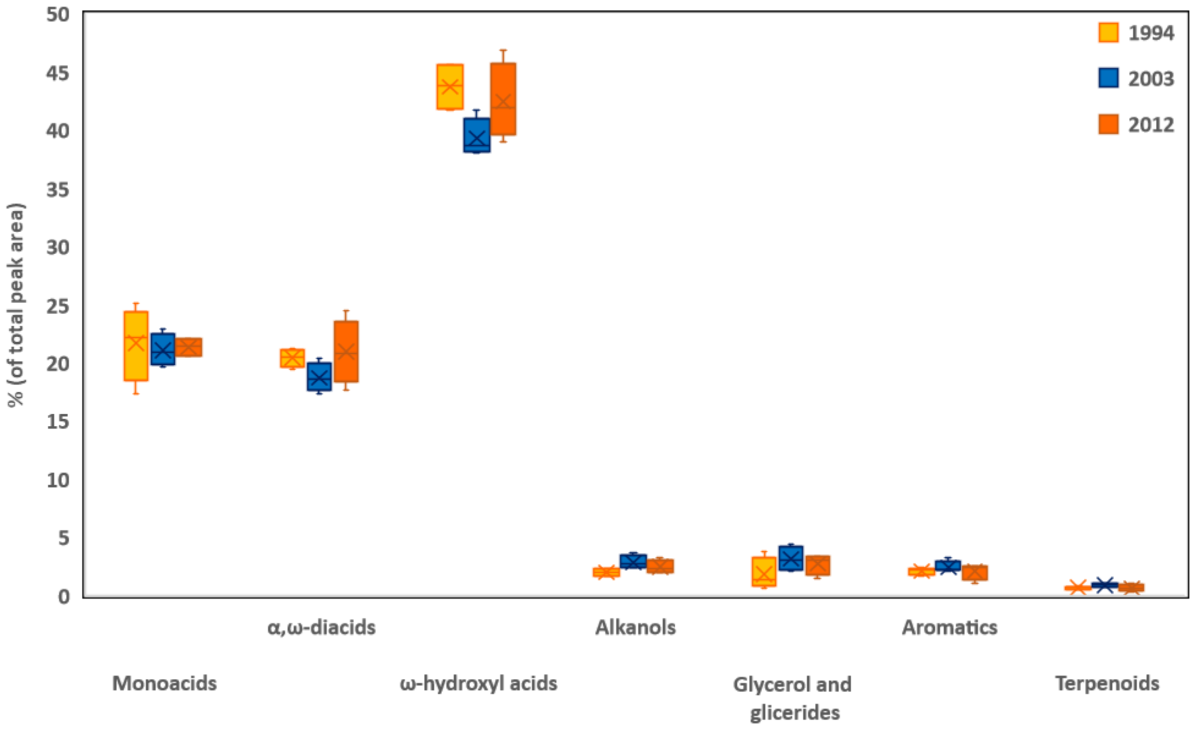

Figure S4 – Monomeric composition of suberin from cork produced without any drought (1994), with two drought events (2003) and one drought event (2012), determined in the GC–MS chromatograms of the depolymerization extracts (% of total peak area).

Table S1 – Composition of suberin from cork produced without any drought (1994), with two drought events (2003) and one drought event (2012), determined in the GC–MS chromatograms of the depolymerization extracts (percentual peak area).

Compound number	Retention time (min.)	Compound	Peak area (%)<br>1994<br>(no drought)	2003<br>(two droughts)	2012<br>(one drought)
**11**	5.14	Glycerol	0.47	0.47	0.24
**17**	6.90	9-Oxononanoic acid methyl ester	0.08	0.06	0.07
**21**	8.31	Vanillin	0.09	0.06	0.08
**27**	10.34	Azelaic acid	0.10	0.12	0.11
**30**	11.89	Isovanillic acid	0.03	0.03	0.03
**31**	12.89	Ferulic acid	0.18	0.17	0.16
**32**	14.42	Hexadecanoic acid methyl ester	0.04	0.04	0.05
**33**	14.99	Ferulic acid methyl ester	1.72	2.05	1.72
**35**	17.41	9-Octadecenoic acid methyl ester	0.05	0.05	0.06
**36**	18.45	Octadecanoic acid methyl ester	0.03	0.03	0.04
**37**	20.18	Hexadecanedioic acid dimethyl ester	2.04	1.55	1.85
**38**	20.72	Hexadecanoic acid, 16-trimethylsiloxy, methyl ester	0.99	0.89	0.96
**41**	21.96	Eicosanol	0.14	0.14	0.14
**42**	22.28	16-Hydroxyhexadecanoic acid methyl ester	0.29	0.58	0.57
**43**	22.79	16-Hydroxyhexadecanoic acid	0.13	0.13	0.14
**44-45**	23.12	9-Octadecenodioic acid dimethyl ester	4.59	3.35	5.10

Compound number	Retention time (min.)	Compound	Peak area (%)<br>1994<br>(no drought)	2003<br>(two droughts)	2012<br>(one drought)
**46**	23.62	18-hydroxy- 9- octadecenoic acid methyl ester	13.23	11.40	13.13
**50**	25.04	Docosanoic acid methyl ester	0.70	0.63	0.71
**52**	25.29	Octadecandioic acid methyl ester	0.84	1.30	1.68
**53**	25.48	Docosanol	0.44	0.54	0.50
**54**	25.71	Oleoacylglicerol	1.18	2.42	2.23
**55**	25.81	Octadecanodioic acid methyl ester	0.13	0.12	0.16
**56**	26.16	Tetradecanoic acid dimethyl ester	0.75	0.66	0.81
**57**	26.58	Oxiraneoctanoic acid	2.25	1.98	2.34
**58**	26.63	9,10-Epoxy-18-hydroxy-octadecanoic acid methyl ester	1.32	1.10	1.08
**59**	26.89	3-Octyl-oxiraneoctanoic acid methyl ester	9.59	8.29	9.48
**60-61**	27.03	9,10-Epoxy-18-hydroxyoctadecanoic acid methyl ester	9.55	8.74	9.36
**62**	27.29	Eicosanodioic acid dimethyl ester	0.85	0.67	0.82
**64**	27.73	Monomyristin	0.16	0.27	0.25
**68**	27.96	Tetracosanoic acid methyl ester	1.06	1.40	1.32
**70**	28.73	Tetracosanol	1.33	1.98	3.15
**71**	28.88	18-Hydroxy-9-octadecenoic acid methyl ester	0.82	1.34	1.43
**72**	29.02	9,10- dihydroxy-octadecanedioic acid dimethyl ester	7.27	6.19	5.62
**74**	29.25	9,10,18-trihydroxid-octadecanoic acid methyl ester	6.45	7.02	5.48
**82**	29.53	Docosanedioic acid dimethyl ester	3.04	2.07	2.52
**83**	30.75	8,9,18-trihydroxyoctadecanoic diacid methyl ester	1.56	2.61	2.31
**84**	30.98	22-Hydroxydocosanoic acid methyl ester	14.62	12.12	13.33

Compound number	Retention time (min.)	Compound	Peak area (%)<br>1994<br>(no drought)	2003<br>(two droughts)	2012<br>(one drought)
**90**	31.21	Hexacosanol	0.11	0.19	0.14
**93**	32.04	Tetracosanoic acid	0.70	0.93	0.98
**95**	32.47	Docosanoic acid	0.96	2.13	1.64
**99**	33.48	Tetracosanodioic acid	0.36	0.28	0.30
**101**	33.85	24-Hydroxy-tetracosanoic acid methyl ester	2.83	2.33	1.97
**105**	34.17	24-Hydroxy-tetracosanoic acid	0.17	0.33	0.21
**110**	35.66	Sitosterol	0.10	0.11	0.11
**111**	38.02	Betulin	0.32	0.56	0.31
**112**	40.86	Betulinic acid	0.06	0.07	0.04

Table S2 – Tree (random factor) variance and residual variance associated to each mixed model, adjusted for extractives, suberin and lignin determinations

Chemical parameter (model)	Tree variance	Residual variance
**Extractives total**	2.00	0.75
**Dichloromethane**	0.54	0.42
**Ethanol**	0.99	0.36
**Water**	0.28	0.48
**Suberin**	13.74	0.53
**Lignin total**	7.79	0.17
**Klason**	7.67	0.15
**Acid soluble**	0.05	0.01
**Ratio suberin/total lignin**	0.07	$6.7x10^{-3}$
